# The Wnt antagonist sFRP1 is downregulated in premalignant large bowel adenomas

**DOI:** 10.1038/sj.bjc.6602967

**Published:** 2006-03-07

**Authors:** G M Caldwell, C E Jones, P Taniere, R Warrack, Y Soon, G M Matthews, D G Morton

**Affiliations:** 1 Division of Medical Sciences, School of Medicine, The University of Birmingham, Birmingham B15 2TH, UK

**Keywords:** colorectal carcinogenesis, frizzled related protein, Wnt signalling

## Abstract

Our previous studies have implicated the Wnt antagonist, sFRP1, as a tumour suppressor gene in advanced colorectal cancer. In this study, we set out to investigate the relationship between sFRP1 expression and large bowel adenomas, a precursor of colorectal cancer. The induction of *β*-catenin/TCF mediated transcription is both a frequent early event in colorectal neoplasia, and a key downstream effect of wnt growth factor signalling. Lithium treatment of a small bowel mucosal cell line (FHs 74 int) induced *sFRP1* within 8 h, indicating that this gene is positively regulated by *β*-catenin, contrasting with the suppression of *sFRP1* expression, we saw previously in advanced colorectal cancers. We therefore investigated a series of 12 adenomas and matched large bowel mucosa samples. Real-time RT–PCR analysis showed a reduction in *sFRP1* expression in all 12 dysplastic lesions (median 485-fold, IQR 120- to 1500-fold), indicating factors other than *β*-catenin influence *sFRP1* levels. In a second series of 11 adenomas, we identified methylation of the sFRP1 promotor region in all 11 samples, and this was increased compared with the surrounding normal mucosa in seven cases. Immunohistochemical analysis using a polyclonal antibody supported these findings, with sFRP1 expression reduced in many of the adenoma samples examined. sFRP1 staining in normal mucosa adjacent to the dysplastic tissue was also reduced compared with the normal controls, suggesting that sFRP1 expression may be suppressed in a field of mucosa rather than in individual cells. This study identifies sFRP1 inactivation at the premalignant stage of colorectal cancer development, indicating that these pathways may be useful targets for chemoprevention strategies in this common solid tumour.

Loss of control over *β*-catenin/TCF-mediated transcription is a central feature of most colorectal tumours (Morin *et al*, 1997). Wnt growth factor signalling can also mobilise *β*-catenin, but its relationship with colorectal tumorigenesis remains unclear. We previously showed (Caldwell *et al*, 2004) that the secreted Wnt antagonist sFRP1 is downregulated in most established colorectal cancers and that this coincides with increased methylation of the gene. This was confirmed in a separate study (Suzuki *et al*, 2004), which also demonstrated similar behaviour in other sFRP family members.

Inactivation of sFRP1 has now been observed in a wide range of malignancies, including ovarian (Takada *et al*, 2004), bladder (Stoehr *et al*, 2004), mesothelioma (Lee *et al*, 2004), breast (Ugolini *et al*, 2001; Klopocki *et al*, 2004), oesophageal (Zou *et al*, 2005), prostate (Lodygin *et al*, 2005) and gastric cancers (Byun *et al*, 2005).

These effects do not appear to be restricted to the sFRPs, however, as other secreted antagonists of Wnt signalling behave in a similar manner. Suppression of WIF-1 expression has been demonstrated in prostate, breast, lung and bladder cancer (Wissmann *et al*, 2003), and this has been attributed to hypermethylation in lung (Mazieres *et al*, 2004) and gastrointestinal cancers (He *et al*, 2005; Taniguchi *et al*, 2005), while DKK-1 expression is also lost in colon cancer (Gonzalez-Sancho *et al*, 2005).

*β*-Catenin-dependent Wnt signalling can induce expression of WIF-1 (He *et al*, 2005) and DKK-1 (Gonzalez-Sancho *et al*, 2005), but this has not been investigated for the sFRPs. It would be reasonable to expect sFRPs to also be induced in response to *β*-catenin, as part of a negative feedback process to desensitise a cell to prolonged Wnt stimuli, but this has not been investigated. In colorectal cancer, at least, the high frequency of APC and other mutations that stabilise *β*-catenin might, therefore, be expected to lead to induction of these Wnt antagonists, including the sFRPs, if they were not suppressed by other mechanisms before the onset of tumorigenesis. Their frequent downregulation therefore indicates that soluble Wnt antagonists either act as tumour suppressors whose expression must be lost at some stage in the development of a range of tumour types, or that their expression is lost coincidentally during tumorigenesis.

Aberrant crypt foci (ACFs) may be the earliest detectable stage of colorectal tumorigenesis, and these have been shown to contain methylated sFRP1 alleles (Suzuki *et al*, 2004). This infers that transcription is suppressed at this early stage, but the precise relationship between sFRP1 methylation patterns and gene expression has not been determined and the expression level cannot be measured in ACFs.

The purpose of this study was to examine the *β*-catenin dependence of sFRP1 and determine its expression in early colorectal tumorigenesis. We investigated the capacity of *β*-catenin to regulate sFRP1 transcription in a normal intestinal epithelial cell line and measured its relative expression and methylation in premalignant colorectal adenomas, the earliest stage of tumorigenesis where expression can be measured reliably. We have also complemented these data with immunohistochemical detection of the sFRP1 protein in a similar adenoma series.

## MATERIALS AND METHODS

### Lithium treatment of cultured cells

The normal small intestine epithelial cell line FHs 74 Int (ATCC) was cultured using Hybri-Care medium (ATCC) supplemented with 1.5g l^−1^ sodium bicarbonate and 10% foetal calf serum. Cells were grown in 6 cm dishes and treated with 10 mM LiCl or 10 mM KCl as a control. Cells were harvested over a time course of 16 h and RNA, DNA and protein were extracted using TRI reagent (Sigma, Gillingham, Dorset, UK).

### Tumour samples

Two cohorts of sporadic adenoma samples were identified from our tumour bank for methylation and expression analysis. All samples were collected from patients over 50 years of age and without a family history of colorectal cancer. All samples were collected from the sigmoid colon or the rectum and matched rectal biopsies were taken at the same procedure (5 cm or more from the tumour site). Samples were collected according to our local ethical committee regulations, with individual consent from each patient for molecular studies. Separate ethical approval was given for analysis of paraffin blocks for protein studies (LREC No. 2003/277).

### Semiquantitative real-time PCR

Total RNA was extracted using TRI reagent (Sigma). First-strand cDNA was synthesised from 2 *μ*g of DNase-treated total RNA using Ready-To-Go You-Prime First-Strand Beads (Amersham Pharmacia Biotech UK Ltd, Little Chalfont, Bucks, UK) and random hexamers (Promega, Southampton, UK).

Oligonucleotide primers and TaqMan probes were designed using Primer Express™, version 1.5 (PE Applied Biosystems, Cambridge, UK). The primers for *sFRP1* (NM_003012) gene amplification were 5′-CCAATCCCACCGAAGCCT and 5′-ATGATGGCCTCAGATTTCAACTC. The sequence for the TaqMan fluorogenic probe was 5′-CAAGCCCCAAGGCACAACGGTG. Data for the *sFRP1* gene were normalised to the epithelial cell-specific gene keratin 8 (*KRT8*) (NM_002273). For *KRT8,* the primers and probe were 5′-GATCGCCACCTACAGGAAGCT, 5′-ACTCATGTTCTGCATCCCAGACT and 5′-CCGGCTCTCCTCGCCCTCCA, respectively. The TaqMan Universal PCR Master Mix and the TaqMan probes were purchased from PE Applied Biosystems. Primers were obtained from Alta Biosciences, Birmingham, UK (University of Birmingham). Multiplex PCR amplifications were performed using an ABI PRISM 7700 Sequence Detector in a final volume of 25 *μ*l. Each reaction contained 12.5 *μ*l of × 2 TaqMan Universal PCR Master Mix (PE Applied Biosystems), 90 nM *KRT8* and *sFRP1* primers, 150 nM *sFRP1* TaqMan probe and 175 nM KRT8 TaqMan probe, 1 *μ*l of cDNA sample and water. The thermal cycling conditions comprised an initial step at 50°C for 2 min and 95°C for 10 min, followed by 40 cycles at 95°C for 15 s and 60°C for 1 min.

### Analysis of promoter methylation status

The method used for DNA modification was essentially that of Grunau *et al* (2001). In short, 10 *μ*g tRNA (Sigma) was added to 1 *μ*g of genomic DNA and made up to 100 *μ*l. Freshly prepared NaOH (Sigma) was added to a final concentration of 0.3 M and the sample was incubated at 42°C for 20 min. In total, 1.2 ml of 5.2–5.69 M sodium bisulfite (Sigma), 10 mM hydroquinone (Sigma) pH 5 was added, the solution overlaid with mineral oil and incubated at 55°C for 4 h. DNA was desalted and re-dissolved into 100 *μ*l of Tris-Cl pH 8 (Sigma). NaOH was added to a final concentration of 0.3 M and the solution was then incubated at 37°C for 20 min. The solution was neutralised, 10 *μ*g of tRNA were added and nucleic acids precipitated with ethanol at –20°C overnight. Precipitated DNA was washed with 70% ethanol, dried and re-suspended in 50 *μ*l of 1 mM Tris-HCl, pH 8.

Methylation status was analysed using the COBRA (COmbined Bisulfite Restriction Analysis) method (Xiong and Laird, 1997). The *sFRP1* CpG island region was predicted using CpG plot (www.ebi.ac.uk). A strong CpG island (island size>100 bp, GC percent>50.0, Obs/Exp>0.6) was detected within the region −180 bp to +530 bp relative to the transcription start site of the *sFRP1* gene. To analyse this region of the gene for methylation, PCR primers specific for the bisulfite modified sequence were designed. The *sFRP1* CpG island was amplified using the primers: *sFRP1* COBRA F 5′-GGTTAGTAGTTGGGTGTTTTTGTTTA and sFRP1 COBRA R 5′-CCTTACCTTAAAACTTAAAAACTTC. One two hundred and fiftieth of this product was then used in a subsequent nested PCR reaction using the primers: *sFRP1* COBRA F-nested 5′-TTGGGTGTTTTTGTTTAATAAGAATT and *sFRP1* COBRA R-nested 5′-AAAACTTATCACACTTAAACATCTC. The PCR conditions used in both reactions were 94°C for 3 min; 30 cycles of 94°C for 20 s, 54°C for 30 s, 72°C for 40 s; 72°C for 7 min and 35 cycles were used in the nested PCR reaction. PCR products were incubated with restriction enzyme *Taq1* for 2 h at 65°C to assay for methylation and visualised on a 2% agarose gel. The COBRA assay for sFRP1 measured the methylation state of the CpG at position +454 from the start codon ATG.

### Immunohistochemistry

An sFRP1 polyclonal antibody was raised in rabbit using a synthetic peptide (GPYQSGRFYTKPPQC) corresponding to amino acids 43–57 of Human sFRP1 (Eurogenetec).

Immunohistochemistry was performed using the streptavidin–biotin indirect immunoperoxidase method as previously described (Hardy *et al*, 2002). Briefly, 5 *μ*m sections were dewaxed, rehydrated and blocked by 10% H_2_O_2_ in methanol for 10 min. Sections were incubated overnight at 4°C with primary antibodies recognising *β*-catenin (clone 14, BD Transduction Laboratories, Cowley, UK) or sFRP1 at a dilution of 1 : 300 and 1 : 1000 respectively. After washing with PBS, sections were incubated with 1 : 500 dilution of biotinylated goat anti-mouse/rabbit (Dako, Ely, UK) for 30 min. Serial PBS washing and incubation with streptavidin-peroxidase conjugate (Dako) was undertaken prior to incubation with diaminobenzidine tetrahydrochloride (Sigma). Sections were counterstained with haemalum (BDH), dehydrated, and then analysed by light microscopy.

Two independent observers (CJ and PT) scored the slides. The number of tumour cells stained was scored as a percentage of the total number of cells (average of at least 10 high power fields). The staining was then reported as strong (>50% cells stained), moderate (10–50%), mild (<10%) or no staining. Any discrepancies between the two observers were then reviewed jointly to reach a final assessment.

## RESULTS

### Effect of *β*-catenin stabilisation on sFRP1 mRNA expression

The transcriptional control of sFRP1 has not been investigated previously. Since it was possible that the frequent downregulation we observed previously in colorectal cancers could be an outcome of *β*-catenin action, we chose to investigate this in Fhs74Int, a non-neoplastic epithelial cell line derived from the small bowel, as no normal colon cell lines are available. Lithium chloride is an inhibitor of GSK3*β* and its consequent ability to stabilise *β*-catenin has been exploited widely to mimic Wnt signalling since this property was characterised (Klein and Melton, 1996). One advantage of using lithium is that it acts downstream of the receptor so, unlike treatment with individual Wnt growth factors, does not depend on the range of FZDs expressed in the cell.

**Figure 1:**
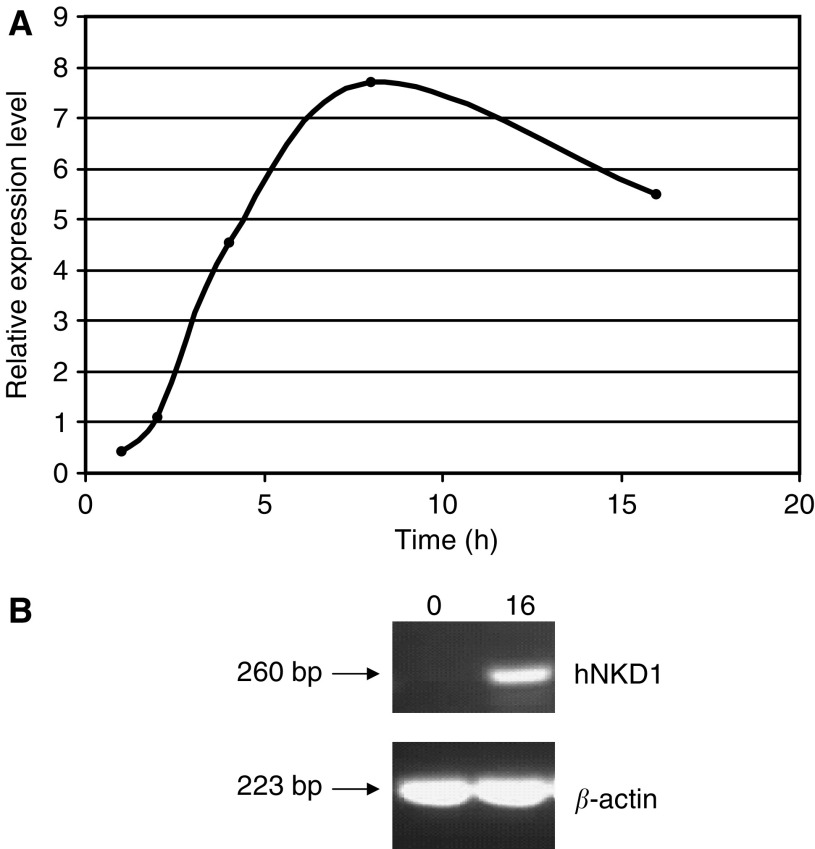
Real-time PCR quantitation of sFRP1 expression levels in a normal cell line following treatment with LiCl. (**A**) Real-time PCR quantitation of relative *sFRP1* mRNA expression measured across a series of time points following lithium chloride treatment of the normal cell line FHS 74 Int according to the comparative *C*_T_ method. The initial step in the calculations is the normalisation of the sFRP1 gene to the KRT8 gene in order to normalise quantity and quality of the cDNA samples. The level of sFRP1 expression at each time point was then normalised to the level of sFRP1 expression before treatment. sFRP1 mRNA levels increased upon LiCl treatment peaking with an eight-fold increase at 8 h, which fell to six-fold at 16 h. (**B**) RT–PCR results for hNKD1 and *β*-actin at time 0 and 16 h.

Cultures were grown to confluence then treated with lithium chloride and sampled over a 16-h time course. *β*-Catenin stabilisation was verified by two approaches. Immunohistochemistry showed nuclear *β*-catenin in cells treated with lithium chloride for 8 and 48 h (result not shown), but not in untreated control cells. We also investigated transcript levels of *NKD1*, a *β*-catenin-dependent gene (Yan *et al*, 2001) by both RT–PCR (Figure 1) and real-time RT–PCR, which showed an increase of over 100-fold after 8 h, confirming induction of *β*-catenin/TCF activity. *sFRP1* transcripts were also induced significantly, to eight times after 8 h, falling to six-fold at 16 h (Figure 1).

These results indicate that rather than suppressing *sFRP1* expression, *β*-catenin/TCF activity is associated with the induction of *sFRP1*, consistent with a feedback response restricting the exposure of a normal cell to a prolonged Wnt growth factor signal. It is unlikely, therefore, that catenin-mediated transcription is responsible for the downregulation of *sFRP1* expression seen in colorectal cancer, indicating that this is caused by an independent event in early colorectal tumorigenesis. Large bowel adenomas are known to be precursors to the majority of colorectal cancers, and are associated with *β*-catenin/TCF signalling. We selected a series of sporadic, left sided, large bowel adenomatous polyps with matched normal mucosa for analysis.

### sFRP1 mRNA expression by real-time quantitative PCR

In order to determine the level of *sFRP1* transcription in early colorectal tumours, we performed quantitative analysis of *sFRP1* mRNA levels, using real-time RT–PCR, in a cohort of 12 adenoma samples and compared these with expression levels in normal bowel mucosa sampled from the same patient. The *sFRP1* expression levels were compared with cytokeratin 8 (*KRT8*) gene expression, providing an epithelial cell-specific marker in order to control for the influence of variation in stromal and inflammatory cell components between dysplastic and normal epithelium. The expression levels of *KRT8* were consistent between adenoma (median Ct value=20.1, IQR 19.7–20.9) and normal samples (median Ct value=20.6, IQR 19.4–21.2).

**Figure 2:**
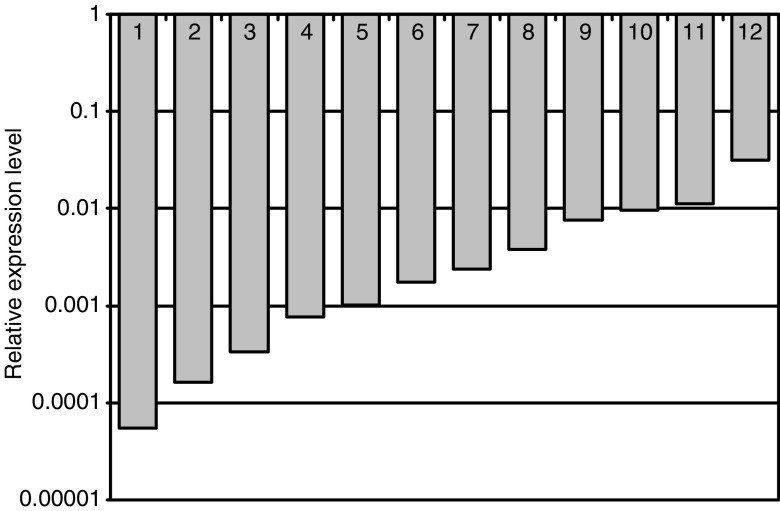
Real-time PCR quantitation of relative sFRP1 mRNA expression in a series of 12 matched colorectal adenoma samples. Real-time RT–PCR was performed and the level of sFRP1 expression in each adenoma sample was compared to the results obtained for the adjacent matched normal tissue. The sFRP1 values are shown on a logarithmic scale. In all 12 adenoma samples, the sFRP1 mRNA levels were reduced substantially (>10 fold) with 83% of the samples showing a reduction of more than a 100-fold.

*sFRP1* mRNA expression was downregulated by more than 10-fold in all 12 adenomas compared with matched normal mucosa (median 485-fold, IQR 120- to 1500-fold, Figure 2). The clinical and pathological data from these 12 adenomas were investigated, but no correlation was found between *sFRP1* expression level, patient sex, tumour size or grade of dysplasia.

These data indicate that *sFRP1* expression is widely and substantially suppressed in premalignant lesions to a level comparable with that seen in advanced cancers of the large bowel and implicates suppression of *sFRP1* expression in the process of premalignant colorectal tumorigenesis. Our previous studies demonstrated that deletion of the sFRP1 gene in early colorectal tumours is a rare event, and we therefore postulated that methylation of the promotor region suppresses expression, as we have previously identified to be the case in advanced colorectal cancer.

### Hypermethylation of the sFRP1 promotor region in colorectal adenoma DNA

**Figure 3:**
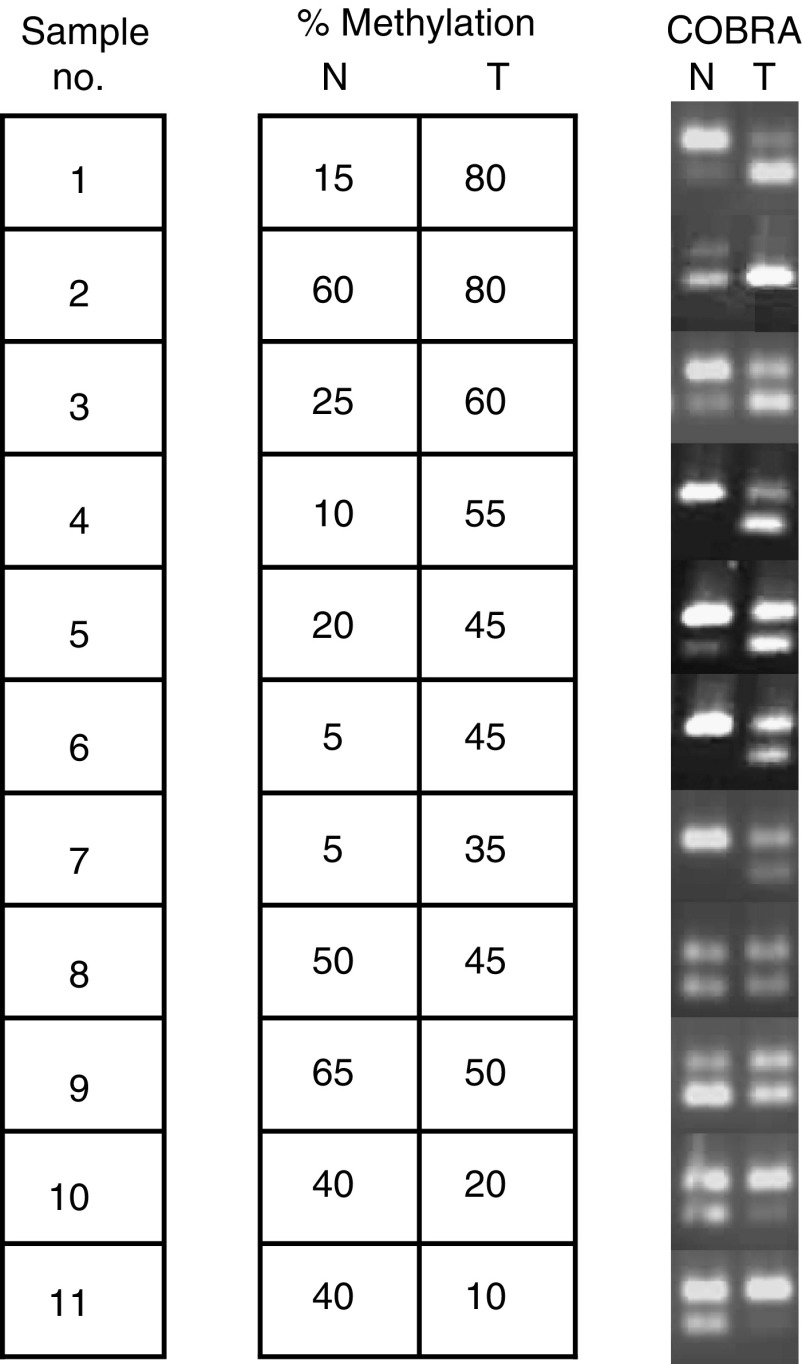
sFRP1 methylation status was analysed using the COBRA (COmbined Bisulfite Restriction Analysis) method as described previously (Caldwell *et al*, 2004). Methylated DNA remains capable of cleavage while unmethylated DNA is resistant. In total, 11 matched colorectal adenoma samples were analysed. The percentage of methylated DNA in each sample was measured by comparing the intensity of the ‘cut’ band against that of the ‘uncut’ band.

To investigate epigenetic changes in the promotor region of sFRP1 in premalignant colorectal lesions, a second cohort of 11 adenoma samples, with matched normal mucosa was selected for analysis by COBRA (COmbined Bisulfite Restriction Analysis) (Xiong and Laird, 1997). This assay measured the methylation state of the CpG at position +454 from the start codon of sFRP1. Methylation was detected in all 11 adenoma samples (Figure 3, median 45%, IQR 35–60%) and hypermethylation of the adenoma, compared with the matched normal mucosa, was seen in seven out of 11 samples. The percentage methylation was unchanged in two matched samples and was reduced in two samples compared to matched normal mucosa. Overall, the level of methylation was higher in adenomas (median 45%) than in normals (median of 25%, IQR-10–50%).

Since the completion of these experiments Suzuki *et al* (2004) have reported sFRP1 promotor hypermethylation in ACFs from large bowel mucosa. That sFRP1 is hypermethylated in both of these lesions lends weight to the hypothesis that ACFs are indeed precursors of large bowel adenomas.

### Immunohistochemical analysis of sFRP1 protein expression and cellular distribution

**Figure 4:**
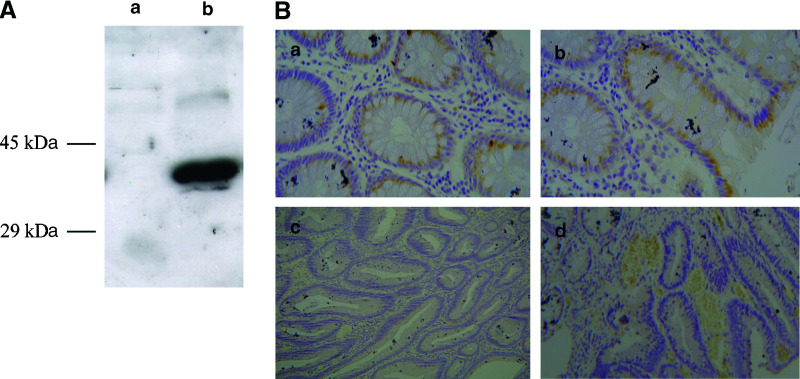
Immunohistochemistry and Western blot analysis using a Polyclonal sFRP1 antibody. (**A**) A Western blot containing lysate from HEK293 cells transiently transfected with full-length mouse sFRP1 and untransfected HEK293 cells was probed an sFRP1 antibody raised in rabbit. The antibody gave a single band at 37 kDa in the transfected HEK293 cell line (b) but no band in untransfected cell (a). (**B**) The same antibody was used to analyse sFRP1 protein expression and cellular distribution in a series of formalin fixed wax embedded sections taken from normal bowel mucosa and colorectal adenomas. All of the normal sections showed moderate to strong cytoplasmic, supranuclear staining (a and b) whereas 45% (10 out of 22) of the adenomas demonstrated either no staining (c) or mild staining (d).

To assess how the expression changes were reflected in the protein level and distribution, we raised a rabbit polyclonal antibody using a synthetic peptide (GPYQSGRFYTKPPQC) corresponding to amino acids 43–57 of human sFRP1. The specificity of the antibody was analysed against HEK293 cells, transiently transfected with mouse sFRP1 by Western blotting. The antibody gave a single band at 37 kD in the transfected HEK293 cell line but no band in untransfected cells (Figure 4).

sFRP1 protein expression and cellular distribution were analysed in a series of formalin-fixed wax-embedded sections taken from seven non-neoplastic large bowel mucosa samples and 22 left sided colorectal adenomas. All of the normal sections stained for sFRP1, four moderately (10–50% of the cells stained) and three strongly (>50% of cells stained). Staining was predominantly in the cytoplasm, and localised to the supranuclear region in both the normal and dysplastic mucosa. Cells stained uniformly at the base and apices of the crypts in normal epithelium.

In contrast to the normal control samples, only nine out of 22 adenoma sections showed moderate staining (10–50% of the tumour cells stained), with three out of 22 demonstrating strong staining (>50% of tumour stained). There was no apparent staining for sFRP1 in five of the adenomas and a further five adenomas showed only mild staining (up to 10% of the tumour cells stained) (Table 1). This represented a reduction in sFRP1 protein expression in at least 45% of these benign adenomas compared with matched controls.

Morphologically normal mucosa was present adjacent to the adenoma in all 22 cases. There was a notable reduction in sFRP1 expression in these normal crypts compared with the normal (non-neoplastic) control samples (*N*=7) used in our IHC study, suggesting there may be a field change in sFRP1 expression. This may also explain why higher levels of methylation were observed in the matched normal mucosa in this study than we had previously identified in normal mucosa from patients with advanced cancers (Caldwell *et al*, 2004), which are usually sampled at a greater distance from the neoplasm.

## DISCUSSION

We have shown that sFRP1 transcription can be driven by *β*-catenin in normal intestinal epithelial cells, but that premalignant colorectal adenomas contain greatly reduced levels of sFRP1 compared to matched normal mucosa and this is accompanied by increased methylation at the sFRP1 locus. Immunohistochemistry with an antibody raised against sFRP1 detected a mostly cytoplasmic signal, which was reduced in adenomas.

In our experiments, lithium chloride treatment stabilised *β*-catenin and induced sFRP1 transcription, consistent with a negative feedback loop whose physiological role would be to antagonise a strong or persistent WNT signal. This parallels previous data for WIF-1 (He *et al*, 2005) and DKK-1 (Gonzalez-Sancho *et al*, 2005), and infers that colorectal tumours should express high levels of all three classes of Wnt antagonists as a result of the *β*-catenin stabilising mutations normally present.

In our adenoma series, however, sFRP1 mRNA levels were reduced by a median factor of 485-fold compared with matched normal mucosa. This corresponds to the pattern we observed previously for sFRP1 in established colorectal cancers (Caldwell *et al*, 2004), indicating that expression is suppressed by another, dominant, mechanism. Another similarity with cancers was the common hypermethylation of the sFRP1 locus in adenomas and it is likely that this contributes to the reduction in transcription we observed.

Our COBRA assay provides an indirect measure of the degree of methylation at the sFRP1 locus, but does not necessarily relate directly to functional suppression of the gene. A previous study (Suzuki *et al*, 2004) surveyed the region around the 5′ end of sFRP1 by bisulphite sequencing. Although this showed an association between the degree of methylation and downregulation of transcription in cancers, it did not reveal a direct relationship between expression levels and methylation of any specific CpG sites. This is likely to prove complex and a more extensive study will be required before it is possible to predict the degree of expression from an sFRP1 allele bearing any given methylation pattern.

The consistently high degree of sFRP1 downregulation revealed by our real-time RT–PCR study and the high frequency of hypermethylation, we observed indicate that these changes are well established at an early stage of tumorigenesis, and the similar effects we reported previously in cancers (Caldwell *et al*, 2004) are unlikely to be the result of genetic change during progression.

Analysis of sFRP1 protein expression by immunohistochemistry gave a less consistent picture than our RNA analysis. One disadvantage of analysing premalignant adenomas is the relatively small size of the sample, which restricts the range of analyses that can be performed and we were unable to compare RNA and protein detection on the same adenoma samples. In normal mucosa, our antibody detected a cytoplasmic signal that appeared, at higher resolution, to be concentrated in a supra-nuclear region that could be the Golgi apparatus. Since sFRP1 is a secreted protein, we expected to stain the cell membrane, but a study using an independent anti-sFRP1 antibody found a similar distribution to ours in the epithelial cells of colonic crypts (Oberschmid *et al*, 2004). This, and our control experiments, indicates that the staining is specific. In contrast, however, a recent study detected sFRP1 transcripts in the underlying mesenchymal cells, and not the epithelium, by *in situ* hybridisation (Gregorieff *et al*, 2005). The reasons for this difference are not clear, but the mesenchymal signal was lost upon tumorigenesis, in agreement with our, and other, mRNA studies.

Immunohistochemical analysis of adenomas indicated a reduction in protein expression, but not the consistent downregulation we saw by real-time RT–PCR. This may be a matter of sampling, since these analyses were performed on different series of adenomas or it may reflect essential differences between these approaches: Real-time RT–PCR is a quantitative, albeit relative, analysis while immunohistochemical protocols are designed for sensitivity and show very poor relationships between the level of target protein and the signal obtained. Immunofluorescence may provide a more proportional signal, but this cannot be performed on paraffin embedded specimens, which were the only form available to us in this study.

The frequent downregulation of sFRP1, and other Wnt antagonists (Gonzalez-Sancho *et al*, 2005; He *et al*, 2005), in colorectal carcinogenesis infers that tumour cells retain a requirement for Wnt signalling despite containing high levels of *β*-catenin and the reason for this is not clear at present. Re-expression of sFRP1 in the colorectal cancer cell line HCT116 led to a reduction in nuclear *β*-catenin (Suzuki *et al*, 2004), while inhibition of WNT1 signalling by a function-blocking antibody caused apoptosis in primary colorectal cancer cells and cell lines (He *et al*, 2005). It appears, therefore, that expression of any of a number of soluble Wnt antagonists might lead to apoptosis and tumour cells must suppress expression of these to survive. It is also plausible that *β*-catenin independent outcomes of Wnt signalling contribute to tumour progression. For example, WNT2 dependent, sFRP1 sensitive, activation of c-jun/AP-1 contributes to cell invasion (Le Floch *et al*, 2005), which may explain the requirement for c-jun action in intestinal tumorigenesis in mice (Nateri *et al*, 2005).

The current data on Wnt antagonists in colorectal cancer indicate that their methylation and inactivation may occur before APC action is lost, and two independent studies in mice are consistent with this. First, inactivation of DNA methyltransferases prevented colorectal tumour formation in APC min mice (Eads *et al*, 2002), indicating that methylation of at least some loci is essential for tumorigenesis. Second, inactivation of APC in the intestine of otherwise normal mice rapidly caused high levels of apoptosis throughout the crypts (Sansom *et al*, 2004) and it is possible that one cause of this is induction of soluble Wnt antagonists in the absence of hypermethylation, blocking Wnt action.

If this interpretation is correct, soluble Wnt antagonists may prove to be effective targets in the chemoprevention of colorectal cancer, as preventing their methylation or causing their re-expression should lead to apoptosis in tumour cells. The apparent requirement for the tumour to suppress members of each of three groups of soluble Wnt antagonists indicates that it may only be necessary to restore the expression of a single species to obtain a therapeutically useful effect.

## Figures and Tables

**Table 1:** Results of immunohistochemistry on seven normal bowel mucosa and 22 adenoma samples (TA=tubular adenoma; TVA=tubular villous adenoma)

	**sFRP1 staining**				
**Sample characteristics**	**None**	**Mild**	**Moderate**	**Strong**	**Total**
*Normal*	0	0	4	3	7
					
*Adenoma*					
Histology					
TA	1	3	5	0	9
TVA	4	2	4	3	13
					
Size					
⩽9 mm	2	3	5	2	12
⩾10 mm	3	2	4	1	10
					
Dysplasia					
Mild	4	2	5	3	14
Moderate	1	2	3	0	6
Severe	0	1	1	0	2
